# Exploring NIR Aza-BODIPY-Based Polarity Sensitive Probes with ON-and-OFF Fluorescence Switching in Pluronic Nanoparticles

**DOI:** 10.3390/polym12030540

**Published:** 2020-03-02

**Authors:** Bahar Saremi, Venugopal Bandi, Shahrzad Kazemi, Yi Hong, Francis D’Souza, Baohong Yuan

**Affiliations:** 1 Ultrasound and Optical Imaging Laboratory, Department of Bioengineering, The University of Texas at Arlington, Arlington, TX 76019, USA; bahar.saremi@uta.edu; 2 Joint Biomedical Engineering Program, The University of Texas at Arlington and The University of Texas Southwestern Medical Center at Dallas, Dallas, TX 75390, USA; yihong@uta.edu; 3 Department of Chemistry, University of North Texas, Denton, TX 76203, USA; venu235@gmail.com (V.B.); Shahrzadkazemi@my.unt.edu (S.K.); 4 Department of Bioengineering, The University of Texas at Arlington, Arlington, TX 76019, USA

**Keywords:** aza-BODIPY, environment-sensitive, polarity-sensitive, near-infrared fluorescence imaging, temperature-sensitive, ultrasound switchable fluorescence probe, Pluronic, F-127, F-96, F-68, thermosensitive

## Abstract

Because of their deep penetration capability in tissue, red or near infrared (NIR) fluorophores attract much attention in bio-optical imaging. Among these fluorophores, the ones that respond to the immediate microenvironment (i.e., temperature, polarity, pH, viscosity, hypoxia, etc.) are highly desirable. We studied the response of six NIR aza-BODIPY-based and structurally similar fluorophores to polarity and viscosity for incorporation inside Pluronic nanoparticles as switchable fluorescent probes (SFPs). Based on our results, all of these fluorophores were moderately to strongly sensitive to the polarity of the microenvironment. We concluded that attaching amine groups to the fluorophore is not necessary for having strong polarity sensitive probes. We further studied the response of the fluorophores when embedded inside Pluronic nanoparticles and found that four of them qualified as SFPs. We also found that the switching ratio of the fluorophore-encapsulated Pluronic nanoparticles (I_ON_-to-I_OFF_) is related to the length of the hydrophobic chain of the Pluronic tri-block copolymers. As such, the highest switching ratio pertained to F-68 with the lowest hydrophobic block poly (propylene oxide) (PPO chain of only 30 units).

## 1. Introduction

Fluorescence imaging is a sensitive and noninvasive method for investigating physiological and biomolecular processes in vitro and in vivo. Tuning the excitation light to 650–900 nm (red/near infrared (NIR)) has made fluorescence imaging in centimeter-deep tissue possible [1]. The red/NIR region is suitable for deep in vivo imaging as water and hemoglobin have their lowest absorption, while tissue autofluorescence and scattering are relatively low [1,2,3,4]. Despite the advantages that red/NIR imaging confers, there are a limited number of fluorescent dyes with excitation and emission in this region. Indocyanine green (ICG), one of the cyanine family dyes, remains the only FDA-approved NIR dye currently administered for clinical applications [1].

Environment-sensitive fluorescent probes are capable of responding to changes in the immediate microenvironment. By changing emission characteristics in response to stimuli or cellular conditions such as polarity, pH, viscosity, hypoxia, ions, etc. [5,6], these probes often forewarn of a severe disease (e.g., higher blood viscosity in diabetic patients [5,7] and lower pH and hypoxia in the tumor tissue of cancer patients [8]).

**Figure 1:**
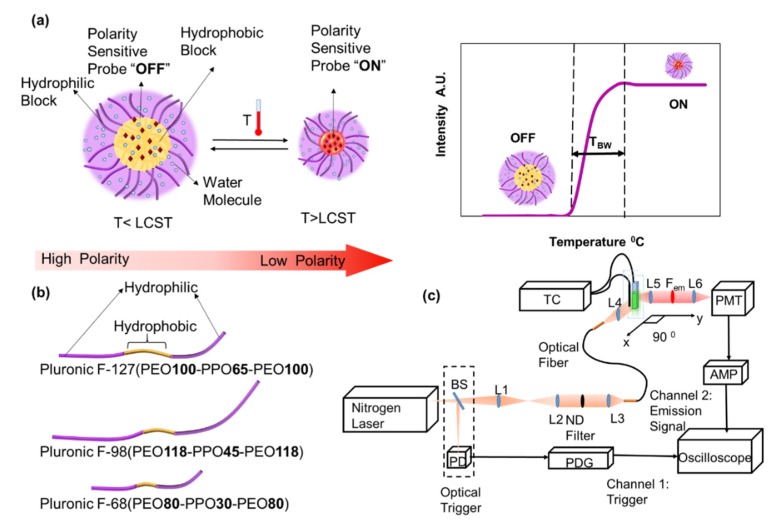
(**a**) Nanoparticles made of thermoresponsive Pluronic block copolymers, embedded with polarity-sensitive fluorophores, respond to the increase in temperature. By shrinking and excreting the water from the core of the nanoparticles, polarity is significantly decreased and polarity-sensitive fluorophores switch to “ON.” (**b**) Pluronic F-127, F-98, and F-68 triblock copolymers with hydrophobic and hydrophilic chains [12,13]. (**c**) A schematic of the optical measurement system.

Among the environmental stimuli, polarity is an important stimulus associated with hydrophobicity of proteins and consequently a broad range of diseases such as Alzheimer’s, in which elevation of hydrophobicity is concurrent with the increase in aggregation-prone proteins [9]. In addition to conveying vital information about the degree of aggregation of proteins, polarity-sensitive fluorescent probes have major applications in the synthesis of thermosensitive switches in conjunction with thermosensitive polymers (Figure 1a).

Although temperature-sensitive fluorescent probes have been reported for numerous applications, they suffer from low sensitivity. Rhodamine-B, a widely used probe for thermometry, has a mild thermosensitivity of 2.3% per degree Kelvin [10]. Multicolor methods, on the other hand, have not attained sensitivities of more than 10% [10]. By contrast, polarity-sensitive fluorescent probes, in conjunction with thermoresponsive polymers, show a dramatic change in fluorescence intensity in response to the change in polarity and, consequently, temperature.

Recently, a new polarity-sensitive fluorophore was introduced, and its characteristics and application as a switchable fluorescent probe (SFP), when incorporated inside polymeric nanoparticles, were studied [11]. In this system, the heat generated from the focused ultrasound would increase the temperature of the tissue at the focus of the ultrasound. Increasing the temperature to above the lower critical solution temperature (LCST) of the probes would elicit a response from the thermoresponsive polymer. The shrinkage of the nanoparticle would affect its water content, and consequently decrease the polarity of the immediate microenvironment of the fluorophores. This would trigger the fluorophore molecules to switch from an “OFF” or dark state, at which they emit weakly, to an “ON” state, at which they emit strongly.

In pursuit of more versatile and stronger environment-sensitive probes, herein, a set of aza-BODIPY-based and structurally related fluorophores were investigated, and their polarity and/or viscosity sensitivity, as well as the structural characteristics leading to such behavior, were explored. The response of the fluorophores was also tested when embedded inside Pluronic nanoparticles (Figure 1a,b).

## 2. Materials and Methods

### 2.1. Fluorophore Characteristics

**Figure 2:**
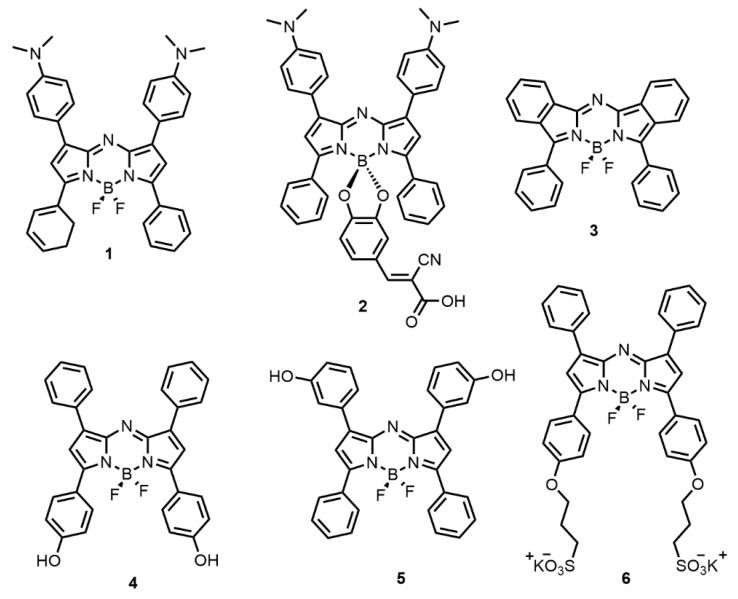
Structure of the aza-BODIPY-based and structurally related fluorophores (synthesis methods have been previously reported for fluorophores 1, 3, 4, 5, 6 [2,14,15,16] and for fluorophore 2 in Supplementary Materials S4).

The optical properties of six aza-BODIPY-based and structurally related fluorophores were investigated in regard to their structure as provided in Figure 2. All fluorophores were synthesized by Dr. D’Souza’s team. The synthesis methods for fluoprophores 1, 3, 4, 5 and 6 have been reported previously [2,14,15,16]. The synthesis method for fluorophore 2 is provided in Supplementary Materials S4. The fluorophores’ names, excitation wavelength (λ_ex_), and detection emission wavelength (λ_em_) are summarized in Table 1. The fluorescence spectra are provided in Supplementary Materials S1.

### 2.2. Fluorescence Measurement System

The fluorescence measurement system utilized for lifetime and intensity measurements was discussed in previous work [17,18], with minor changes to accommodate the temperature control system and corresponding filters for different fluorescent dyes. Briefly, a combined laser system from Optical Building Blocks Corporation (Birmingham, NJ, USA) generated an 800 ps pulse at the excitation wavelength of each dye. All lenses used in the system were purchased from Thorlabs Inc. (Newton, NJ, USA), if not otherwise stated. The generated fluorescence light passed through a converging lens, and the corresponding emission band-pass or long-pass filter (BP, FF01-711/25-25, FF01-785/62-25, or BLP01-808R-25 from Semrock, Rochester, NY, USA). The emitted fluorescent light was detected by a photomultiplier tube (PMT, H10721-20, Hamamatsu, Japan). A pulse delay generator (PDG, DG645, Stanford Research Systems, Sunnyvale, CA, USA) triggered the 2.5 GHz oscilloscope (DPO 7254, Tektronix, Beaverton, OR, USA). The output of the PMT was converted to a voltage signal and was amplified by a broadband preamplifier (C5594, bandwidth from 50 kHz to 1.5 GHz, Hamamatsu, Japan). The signal was ultimately acquired by the multichannel and broadband oscilloscope. Each emission decay pulse recorded from the oscilloscope, used for calculating fluorescence lifetime, was averaged 100 times. A temperature controller (PTC10, Stanford Research System, Sunnyvale, CA, USA) was used to control the temperature (Figure 1c).

### 2.3. Fluorescence Emission Intensity and Lifetime Measurement

Since the fluorescence signal is acquired by an oscilloscope, the peaks of the decay curves indicate the voltage, and are proportional to the fluorescence intensity. For lifetime calculations, the acquired signal and the impulse response function were deconvolved and the decay curve was fitted to a monoexponential decay function [18]. Calculations were done using MATLAB (Natick, MA), with an iterative method to find the best fit with lowest residue. For fluorescence intensity calculations in polarity experiment, after averaging the emission curves 100 times, a moving average filter (n = 5) was applied, and the peak height of the decay curve was obtained in each solvent for all fluorophores. Fluorescence intensity of each fluorophore in water was normalized to 1. For fluorescence intensity calculations in fluorophore-encapsulated Pluronic nanoparticles, the same protocol was followed without a moving average filter. Herein, the peak voltage of the decay curve at each temperature was obtained (mV), and the switching ratio was calculated as discussed in the text. For comparisons regarding the Pluronic nanoparticles, a two-tail *t*-test (equal variance) was conducted. Pearson linear correlation coefficient was calculated with R program.

### 2.4. Solvents with Different Polarities

Fluorescence lifetime and emission intensity of the fluorophores were measured in different solvents. Solvents were obtained from Sigma-Aldrich Corporate (St. Louis, MO, USA), if not otherwise stated. Characteristics of the solvents used are provided in Table 2. The final concentration of fluorophores was kept at 50 nM.

### 2.5. Solvents with Different Viscosities

To cover a range of viscosities, different mixtures of ethylene glycol (EG) and glycerol (Gl) were prepared. Solutions were prepared by mixing Gl and EG at different volume ratios: Gl/EG (*v*/*v*) % of (0/100)%, (16/84)%, (50/50)%, (75/25)%, (92/8)%, and (100/0)%. The viscosity of the mixtures (Table 3) were measured with an A&D SV-10 Viscomter (Tokyo, Japan).

### 2.6. Preparation of Fluorophore-Encapsulated Pluronic Nanoparticles

Nanoparticles were synthesized based on a revised protocol from Pluronic block copolymers consisting of poly(ethylene oxide) (PEO) and hydrophobic poly(propylene oxide) (PPO) [22]. Pluronic F-127 (PEO100-PPO65-PEO100) [12] obtained from Sigma-Aldrich (St. Louis, MO, USA), as well as Pluronic F-98 (PEO118-PPO45-PEO118) [13] and Pluronic F-68 (PEO80-PPO30-PEO80) [13] obtained from BASF (Florham Park, NJ, USA), were dissolved in deionized water (D.I. water) at pH 8.5 with a 5% (*w*/*v*) ratio with stirring. The fluorophores and tetrabutylammonium iodide (TBAI) from Sigma-Aldrich (St. Louis, MO, USA) were dissolved with a molar ratio of 1:8 in 6 mL chloroform (Fisher Scientific, Pittsburgh, PA, USA) by sonication using a bath ultrasonic cleaner (Branson 1510, Branson Ultrasonic Corporation, Danbury, CT, USA) and added drop-wise to 15 mL of the Pluronic solution while stirring at 1200 rpm. The sample was then sonicated with an XL-2020 probe-sonicator (Misonix, Farmingdale, NY, USA), while the probe intensity was kept at ~5–5.5. The sample was then moved to a beaker covered by aluminum foil with generated holes and stirred at 200–300 rpm overnight to evaporate the chloroform (final concentration of fluorophores was kept at about 50 µM). Nanoparticles were then filtered with (10,000 MWCO) Amicon ultra centrifugation filters (Merck Millipore, Billerica, MA, USA) and large particles and/or impurities were filtered by 0.45 µm membrane filters (Fisher Scientific, Pittsburgh, PA, USA). The effect of filtration and dilution on LCST has been extensively studied in the Supplementary Materials S3. For filtration, samples were diluted 5 times (for facilitating the process), centrifuged at 4500 G with a Legend X1 centrifuge (Sorvall™ Legend™ X1, Thermo, Marietta, OH, USA), reduced, and then brought back to the initial volume with D.I. water. All measurements were done with filtered 1% samples, except for when the effect of filtration was studied, where 1% and 0.2% samples, with and without filtering, were prepared.

## 3. Results and Discussion

### 3.1. Response to Polarity

**Figure 3:**
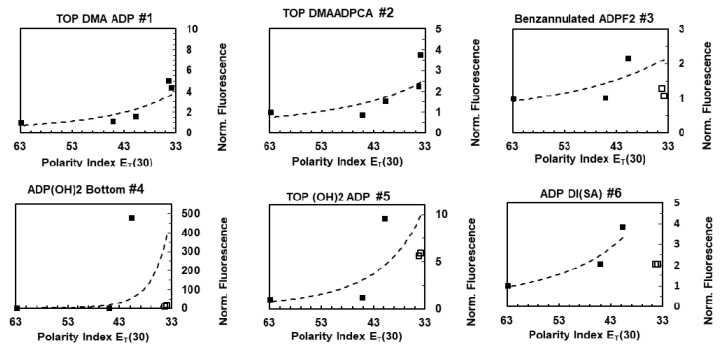
Peak fluorescence emission signal intensity vs solvent polarity. Power function fitted to the points depicted with black squares. The fluorescence intensity in water is normalized to 1.

To investigate the effect of polarity, the fluorescence intensity of the fluorophores, were measured in different solvents. By changing the solvents, from water to toluene, the polarity of the microenvironment changed 28.9 units of polarity index, E_T_ (30). This polarity index was defined as the molar electronic transition energy of the negatively solvatochromic pyridinium N-phenolate betaine dye as probe molecule, measured in kilocalories per mole (kcal mol^−1^) at room temperature (25 °C), and normal pressure (1 bar) [23]. Normalized fluorescence intensity vs polarity index is plotted in Figure 3. The decay curves are provided in Supplementary Materials S2. The maximum emission peak for each fluorophore among solvents is referred to as I_max_, and the emission peak in water is denoted as I_water_.

As demonstrated in Figure 3, based on the normalized fluorescence intensity values, ADP(OH)2 Bottom (i.e., fluorophore 4) had the strongest dependency on the polarity with I_max_-to-I_water_ ratio of ~478, followed by Top (OH)_2_ ADP (i.e., fluorophore 5) with a ratio of ~9.58. Top Dimethyl amine ADPCNCA (TOP DMAADPCA, i.e., fluorophore 2) and Top Dimethyl amine ADPF2 (TOP DMA ADP, i.e., fluorophore 1) had a ratio of 3.77 and 5.04 times, respectively. ADP Di Sulphonic acid (ADP Di (SA), i.e., fluorophore 6) had a ratio of 3.8 times, followed by Benzannulated ADPF2 (i.e., fluorophore 3) that had the lowest ratio of 2.16 times. A previous publication reported that Top Dimethyl amine ADPF2 (TOP DMA ADP, i.e., fluorophore 1) has polarity sensitivity between 677 and 692 nm [14]. Because of the particular interest in the red/NIR region, we measured the polarity sensitivity between 723 and 847 nm, in this study.

The significant decrease in the fluorescence intensity from the highest polarity value, observed for fluorophores 3, 4, 5 and 6 in benzene and toluene (the last two squares) might be due to lower solubility in highly nonpolar solvents. Since we are only interested in the response to polarity, the points in which the fluorescence intensity was affected by solubility were shown with hollow squares and not included in fitting. To limit the effect of solubility, very low concentrations (50 nM) were used, and no aggregation was observed. In general, the fluorescence emission intensity of all the fluorophores increased with the decrease in polarity. The existence of amine groups attached to the fluorophore system seems unnecessary for generating a strong polarity-sensitive probe. As such, fluorophore 4 is a highly sensitive probe, despite not having an amine group.

In the case of fluorophores with substitutions of electron-donating groups to the aryl rings attached to the core of the fluorophore, polarity sensitivity was most prominent when the hydroxyl groups were attached to the para position of aryls at 3- and 5- (fluorophore 4), and to a lesser degree, when hydroxyl groups were attached to the meta position of the aryls at 1- and 7- (fluorophore 5), or when the sulfonate groups were attached to the aryls at 3- and 5- positions (fluorophore 6). More conclusive results were obtained by synthesizing Pluronic nanoparticles embedded with the fluorophores.

### 3.2. Response to Viscosity

The fluorescence intensity and lifetime of the fluorophores were measured in regard to the viscosity of the environment, while polarity was kept relatively constant (below 3.2 units of E_T_ (30)). To quantitatively investigate the relationship between the fluorescence intensity with the viscosity, the Förster–Hoffmann equation was used [24,25]:

log (I) = C + Χ log(ɳ)(1)

**Figure 4:**
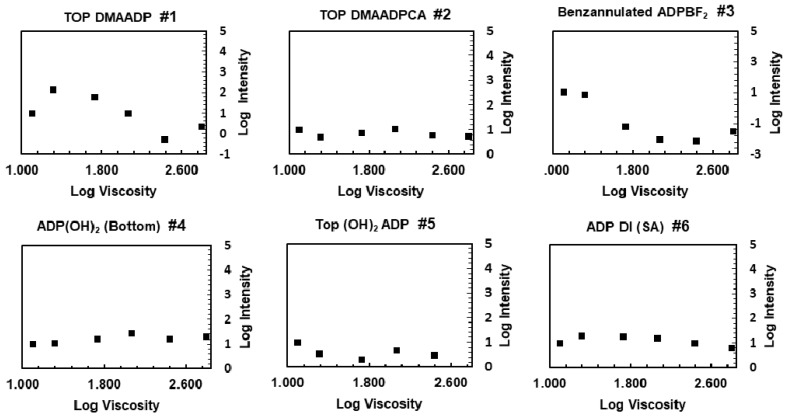
Fluorescence intensity in response to the change in the viscosity.

Here, I is the peak emission intensity, ɳ is the solvent viscosity, C is a constant related to temperature and concentration, and Χ is a constant related to fluorophore properties. For each fluorophore, the logarithm of signal peak in EG (lowest viscosity) was normalized to 1. As evident from the graphs (Figure 4), the fluorescence intensity of fluorophores 2, 4, 5, and 6 was relatively insensitive to viscosity. The fluorescence intensity of ADP BF_2_ (fluorophore 3) dropped in the viscous medium, while the fluorescence intensity of fluorophore 1 increased about 14 times at the beginning, and then decreased. The decrease in fluorescence intensity could be due to lack of a response to viscosity (fluorophore 3), or a response (fluorophore 1) compounded by the decrease in fluorescent emission, caused by the change in polarity in this experiment. It should be noted that by changing the composition of the mixture, polarity changed 3.2 units of E_T_ (30), and inevitably the response to viscosity was convolved with the response to polarity. Although our result does not refute the fact that loss of energy due to rotational motion happens, it implies that viscosity sensitivity is not a major contributor to the phenomenon under study. Our data is consistent with previous studies reported in the literature that phenyl substitutions at 3- and 5- positions cannot rotate freely as a result of the steric hindrance caused by interaction of the hydrogen in the C-H bond with the fluorine atom from the BF2 [26,27].

### 3.3. Fluorophore Encapsulation in Pluronic Nanoparticles

**Figure 5:**
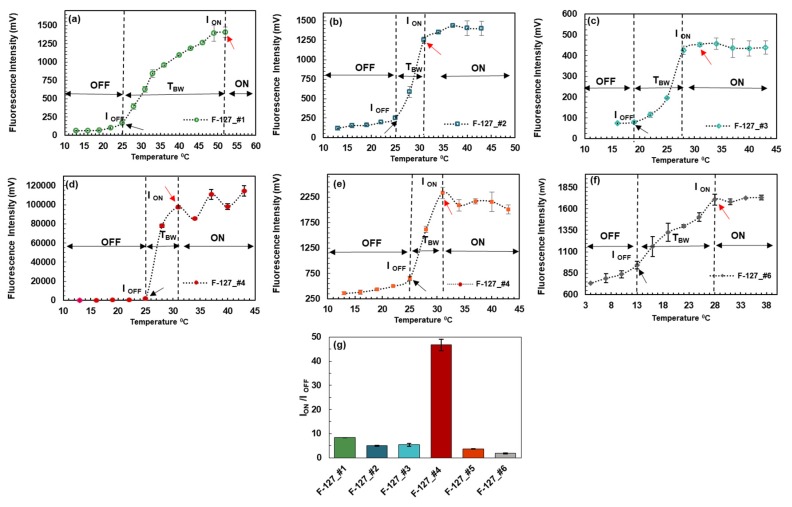
(**a**–**f**) Response of Pluronic F-127 loaded with fluorophores to the change in temperature. Black arrows show the LCST and red arrows the first local maximum (major slope change in the case of a plateau). (**g**) Pluronic F-127 nanoparticles loaded with fluorophore 4 acted as a very strong switch. Both F-127 nanoparticles loaded with fluorophore 2, 3 and 5, act as moderate switches. F-127 nanoparticles loaded with fluorophore 1, and 6 did not qualify as a switch due to having a large *T*_BW_ (≥15 °C). (Values are based on Edge method).

Based on the polarity sensitivity results, while all fluorophores responded to the change in polarity, it was concluded that the attachment of amine groups to the fluorophore was not necessary for having strong polarity-sensitive probes. For the next step, the fluorophores were encapsulated into Pluronic F-127 nanoparticles, and their response to temperature was measured with increasing the temperature (Figure 5). Fluorophore 4 was also encapsulated in Pluronic F-98 and F-68 nanoparticles, to investigate the effect of hydrophobic and hydrophilic chain lengths of Pluronic nanoparticles.

As shown in Figure 5, when the temperature is below the LCST (lower critical solution temperature) of the nanoparticles (black arrow), the fluorescence emission is very weak because the fluorophores in the nanoparticles are exposed to a microenvironment in which water molecules are rich, and therefore, the polarity is high and thus, the fluorophore has a low emission efficiency. When the temperature is above the LCST, the fluorescence emission increases rapidly. Eventually, the fluorescence intensity reaches a plateau (red arrow). This can be understood as follows. When the temperature is above the LCST, the fluorophores in the nanoparticles are exposed to a lack-of-water microenvironment in which the polarity is low, and thus the fluorophore has a high emission efficiency. Herein, the temperature range between the first local maximum and the LCST is the temperature transition bandwidth (*T*_bw_). If the *T*_bw_ is as narrow as a few degrees, the Pluronic-fluorophore system can act as a switch, in which the fluorophore is considered to be at an “OFF” state when temperature is below the LCST, and at an “ON” state when temperature is above the first local maximum.

For calculating the switching ratio (I_ON_-to-I_OFF_), two methods are proposed: (1) The edge method, in which the amplitude of the voltage at the first local maximum (or in the case of a plateau, the first point at major slope change) is divided by the amplitude of the voltage at the LCST, and (2) the vicinity method, in which the amplitude of the voltage at the first local maximum is divided by the averaged amplitude of the voltage of the points at the vicinity of the LCST (the temperature of choice below LCST depends on the application, and 3 °C was chosen here).

As shown in Figure 5 and Table 4, Pluronic F-127 with fluorophore 4 had the highest ratio of switching among all other fluorophores (46.74 times). Since the T_BW_ for this system is 6 °C, this polymer-fluorophore system qualified as a very strong switch.

Despite the (I_ON_-to-I_OFF_) ratio of Pluronic F-127 with fluorophore 1 (8.37 times), this system doesn’t behave as a switch. This is due to the very large *T*_BW_ of more than 27 °C. Pluronic F-127 with fluorophores 2, 3 and 5 with an (I_ON_-to-I_OFF_) of 4.97, 5.4 and 3.59 times, respectively, act as moderate switches. F-127 nanoparticle system with fluorophore 6, has an (I_ON_-to-I_OFF_) of (1.81 times) over a wide bandwidth of 15 degrees and didn’t behave as a switch.

**Figure 6:**
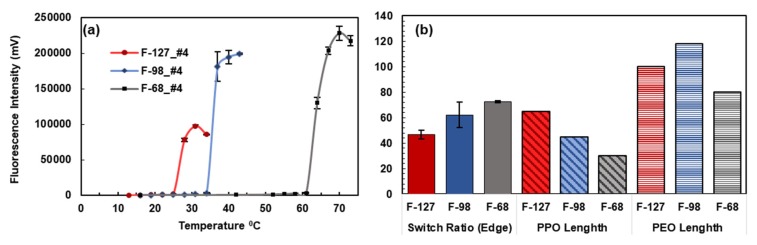
(**a**) Response of Pluronic F-127, F-98, and F-68 nanoparticles loaded with fluorophore 4 to the change in temperature. (**b**) Pluronic F-68 nanoparticles loaded with fluorophore 4 showed a stronger switching ratio compared to Pluronic F-127 nanoparticles (*p*-value of 0.009). The switch ratio is inversely correlated to length of hydrophobic (PPO) chain (*ρ* = −0.99, *p*-value = 0.018).

Compared to F-127, Pluronic F-68 with fluorophore 4 elicits a higher value of I_ON_-to-I_OFF_ of 72.74 times (Figure 6). Based on the Pearson linear correlation coefficient, there was a strong inverse correlation between the switch ratio and the length of the hydrophobic chain of the Pluronic tri-block copolymer. As such, the shorter length of the hydrophobic chain of the Pluronic tri-block copolymer would yield a higher switching ratio (30 PPO units of F-68 compared to the 65 PPO units of F-127). Although future research is needed to delineate this phenomenon, we suspect that the fluorophores are embedded in the hydrophobic region of the micelles, and the shorter length of the hydrophobic chain would produce a less polar environment upon being heated.

The observed significant polarity sensitivity of the fluorophores, considering their interconnected π-system, might be due to specific solvent-fluorophore interactions such as “charge transfer pathways,” which mainly include photo-induced electron transfer (PeT) and internal charge transfer (ICT).

## 4. Conclusions

Six members of the aza-BODIPY-based and structurally related fluorophores with excitation and emission in the red/NIR region were investigated. It was shown that fluorophore 4 was a very strong polarity-sensitive probe, followed by fluorophores 1, 2, 5, 6, and 3. It was also shown that attaching amine groups to the fluorophore is not necessary for having strong polarity-sensitive probes. After encapsulating fluorophores into thermosensitive Pluronic nanoparticles, it was found that fluorophores 4, 2, 3, and 5 can be used as ON-and-OFF fluorescence switches because of the dramatic change in peak emission fluorescence intensity over a narrow range of temperatures (fluorophore 4 had the strongest switching response). It was also shown that the switching ratio of the fluorophore-encapsulated Pluronic nanoparticles (I_ON_-to-I_OFF_) increased with decreasing the number of hydrophobic chains. As such, Pluronic F-68 with a PPO length of 30 had the highest switching ratio, while the Pluronic F-127 nanoparticles with the PPO length of 65 had the lowest.

## Figures and Tables

**Table 1:** Summary of the fluorophores’ names, excitation, and emission detection wavelengths adopted in the experiments [2,14,15,16] and Supplementary Materials S4.

	Fluorophore Name	Abbrev. Name	λ_ex_ (nm)	λ_em_ (nm)
**1**	Top Dimethyl amine ADPF2	TOP DMAADP	644	785/62 BP
**2**	Top Dimethyl amine ADPCNCA	TOP DMAADPCA	644	808 LP
**3**	Benzannulated ADPF2	Fused ADPF_2_	710	785/62 BP
**4**	ADP (OH)_2_ (Bottom)	ADP(OH)_2_(Bottom)	655	711/25 BP
**5**	Top (OH)_2_ ADP	Top (OH)_2_ ADP	655	711/25 BP
**6**	ADP-Di Sulphonic acid	ADP DI(SA)	644	711/25 BP

**Table 2:** Solvent properties [19,20,21].

Solvent	Solvent Type	E_T_(30) (kcal/mol)	Dielectric Constant ε
Water	Polar protic	62.8	80.1
DMSO	Dipolar aprotic	45.1	46.7
Dichloromethane	Polar aprotic	40.7	9
Benzene	Nonpolar	34.3	2.3
Toluene	Nonpolar	33.9	2.4
Glycerol	Polar protic	57	42.5
Ethylene glycol	Polar protic	53.8	31.8

**Table 3:** Viscosities pertaining to Gl/EG (*v*/*v*) % solutions.

**Glycerol %**	0	16	50	75	92	100
**Viscosity (m Pa.s)**	12.7	20.8	53.5	117.0	273.5	634.5

**Table 4:** Summary of switching properties of the fluorophore-encapsulated Pluronic nanoparticles.

Pluronic Nanoparticles	I_ON_-to-I_OFF_ Edge Method	I_ON_-to-I_OFF_ Vicinity Method	LCST * (°C) Nanoparticles	*T*_BW_ (°C)	Lifetime (ns) (OFF to ON Edge Method)
F-127_#1	8.37 ± 0.11	13.74 ± 1.39	25	27	0.41 ± 0.01 to 1.09 ± 0.01
F-127_#2	4.97 ± 0.4	6.36 ± 0.17	25	6	0.27 ± 0.1 to 0.75 ± 0.06
F-127_#3	5.40 ± 0.76	5.75 ± 0.34	19	9	1.42 ± 0.45 to 1.81 ± 0.05
F-127_#4	46.74 ± 3.35	109.1 ± 18.9	25	6	2.0 ± 0.14 to 3.02 ± 0.03
F-127_#5	3.59 ± 0.08	4.65 ± 0.14	25	6	2.55 ± 0.07 to 3.02 ± 0.17
F-127_#6	1.81 ± 0.15	2.04 ± 0.18	13	15	2.96 ± 0.23 to 3.23 ± 0.08
F-98_#4	62.26 ± 9.9	137.23 ± 0.15	34	6	1.5 ± 0.1 to 1.65 ± 0.05
F-68_#4	72.74 ± 0.81	102.07 ± 3.72	61	9	1.14 0.3 to 2.33 ± 0.04

* Lower critical solution temperature.

## Supplementary Materials

The following are available online at https://www.mdpi.com/2073-4360/12/3/540/s1, Supplementary Materials S1: Absorbance and Emission Spectra, Supplementary Materials S2: Fluorescence Lifetime Decay Curves, Supplementary Materials S3: Effect of Filtration and Dilution on LCST, Supplementary Materials S4: Compound 2 (Top DMAADPCA) Synthesis Procedure.

## References

[1] Weissleder R. (2001). A clearer vision for in vivo imaging. Nat. Biotechnol..

[2] Tasior M., Murtagh J., Frimannsson D.O., McDonnell S.O., O’Shea D.F. (2010). Water-solubilised BF2-chelated tetraarylazadipyrromethenes. Org. Biomol. Chem..

[3] Frangioni J.V. (2003). In vivo near-infrared fluorescence imaging. Curr. Opin. Chem. Boil..

[4] Lu H., Mack J., Yang Y., Shen Z. (2014). Structural modification strategies for the rational design of red/NIR region BODIPYs. Chem. Soc. Rev..

[5] Yang Z., Cao J., He Y., Yang J.H., Kim T., Peng X., Kim J.S. (2014). Macro-/micro-environment-sensitive chemosensing and biological imaging. Chem. Soc. Rev..

[6] Klymchenko A. (2017). Solvatochromic and Fluorogenic Dyes as Environment-Sensitive Probes: Design and Biological Applications. Accounts Chem. Res..

[7] Irace C., Carallo C., Scavelli F., De Franceschi M.S., Esposito T., Gnasso A. (2013). Blood Viscosity in Subjects With Normoglycemia and Prediabetes. Diabetes Care.

[8] Kato Y., Ozawa S., Miyamoto C., Maehata Y., Suzuki A., Maeda T., Baba Y. (2013). Acidic extracellular microenvironment and cancer. Cancer Cell Int..

[9] Mitchell M.J., King M.R. (2014). Increased Protein Hydrophobicity in Response to Aging and Alzheimer’s disease. NIH Public Access.

[10] Estrada-Pérez C.E., Hassan Y.A., Tan S. (2011). Experimental characterization of temperature sensitive dyes for laser induced fluorescence thermometry. Rev. Sci. Instrum..

[11] Cheng B., Bandi V., Yu S., D’Souza F., Nguyen K.T., Hong Y., Tang L., Yuan B. (2017). The Mechanisms and Biomedical Applications of an NIR BODIPY-Based Switchable Fluorescent Probe. Int. J. Mol. Sci..

[12] Pitto-Barry A., Barry N.P.E. (2014). Pluronic^®^ block-copolymers in medicine: from chemical and biological versatility to rationalisation and clinical advances. Polym. Chem..

[13] Lee C.-F., Tseng H.-W., Bahadur P., Chen L.-J. (2018). Synergistic Effect of Binary Mixed-Pluronic Systems on Temperature Dependent Self-assembly Process and Drug Solubility. Polymers.

[14] Killoran J., McDonnell S.O., Gallagher J.F., O’Shea D.F. (2008). A substituted BF2-chelated tetraarylazadipyrromethene as an intrinsic dual chemosensor in the 650–850 nm spectral range. New J. Chem..

[15] Gresser R., Hummert M., Hartmann H., Leo K., Riede M.K. (2011). Synthesis and Characterization of Near-Infrared Absorbing Benzannulated Aza-BODIPY Dyes. Chem. A Eur. J..

[16] Bandi V., El-Khouly M.E., Nesterov V.N., Karr P.A., Fukuzumi S., D’Souza F. (2013). Self-Assembled via Metal-Ligand Coordination AzaBODIPY-Zinc Phthalocyanine and AzaBODIPY-Zinc Naphthalocyanine Conjugates: Synthesis, Structure, and Photoinduced Electron Transfer. J. Phys. Chem. C.

[17] Cheng B., Wei M., Liu Y., Pitta H., Xie Z., Hong Y., Nguyen K.T., Yuan B. (2013). Development of Ultrasound-Switchable Fluorescence Imaging Contrast Agents Based on Thermosensitive Polymers and Nanoparticles. IEEE J. Sel. Top. Quantum Electron..

[18] Saremi B., Wei M.-Y., Liu Y., Cheng B., Yuan B. (2014). Re-evaluation of biotin-streptavidin conjugation in Förster resonance energy transfer applications. J. Biomed. Opt..

[19] Haidekker M.A., Brady T., Lichlyter D., Theodorakis E. (2005). Effects of solvent polarity and solvent viscosity on the fluorescent properties of molecular rotors and related probes. Bioorganic Chem..

[20] Web Services at the European Bioinformatics Institute. https://www.ebi.ac.uk/chebi/.

[21] Murov S.L. Properties of Solvents Used in Organic Chemistry. http://murov.info/orgsolvents.htm.

[22] Kim T.H., Chen Y., Mount C., Gombotz W.R., Li X., Pun S.H. (2010). Evaluation of Temperature-Sensitive, Indocyanine Green-Encapsulating Micelles for Noninvasive Near-Infrared Tumor Imaging. Pharm. Res..

[23] Reichardt C. (1994). Solvatochromic Dyes as Solvent Polarity Indicators. Chem. Rev..

[24] Su D., Teoh C.L., Gao N., Xu Q.-H., Chang Y.-T. (2016). A Simple BODIPY-Based Viscosity Probe for Imaging of Cellular Viscosity in Live Cells. Sensors.

[25] Haidekker M.A., Theodorakis E.A. (2010). Environment-sensitive behavior of fluorescent molecular rotors. J. Boil. Eng..

[26] Zhang X., Yu H., Xiao Y. (2011). Replacing Phenyl Ring with Thiophene: An Approach to Longer Wavelength Aza-dipyrromethene Boron Difluoride (Aza-BODIPY) Dyes. J. Org. Chem..

[27] Chen J., Reibenspies J., Derecskei-Kovacs A., Burgess K. (1999). Through-space 13C–19F coupling can reveal conformations of modified BODIPY dyes†. Chem. Commun..

