# Supplementary material for: Exploring NIR Aza-BODIPY-Based Polarity Sensitive Probes with ON-and-OFF Fluorescence Switching in Pluronic Nanoparticles
Source: Polymers (Basel). 2020 Mar 2;12(3):540. doi: 10.3390/polym12030540 (PMC7183047; doi:10.3390/polym12030540)
Supplement: Supplementary file 1 [file polymers-12-00540-s001.pdf]

Supplemental material

S1. Absorbance and Emission Spectra

Absorbance and emission spectra for the fluorophores are provided in **Figure S1**. Spectral measurements for all fluorophores except #1 and #2 were carried out by a Shimadzu (2550 double monochromator) UV-visible spectrophotometer, while the emission was monitored by a Varian Eclipse spectrometer. The spectra of #1 and #2 were measured using a Tecan (SPARK 10M). The excitation wavelengths are provided in **Table 1**.

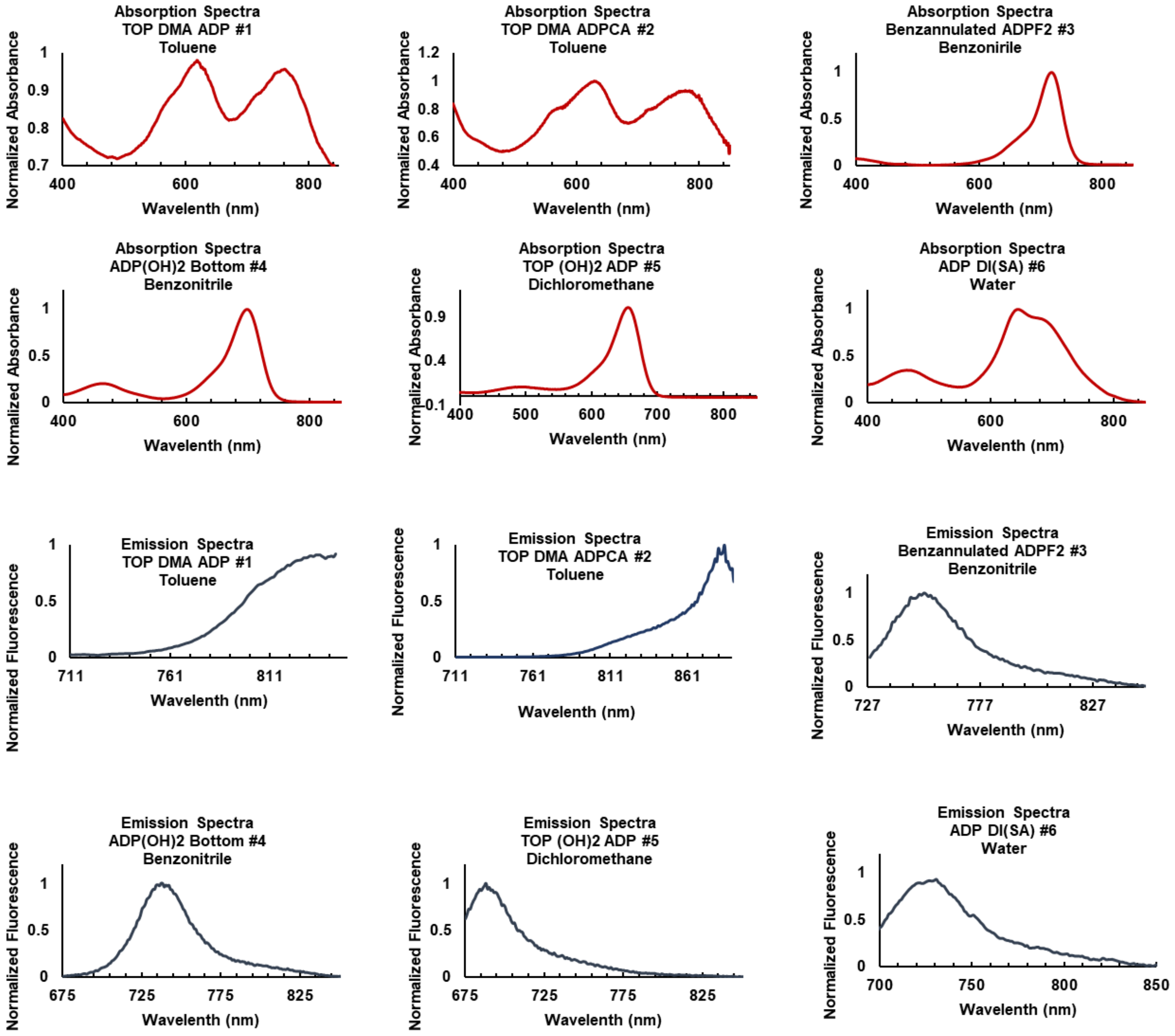

Figure S1. Absorption and emission spectra for fluorophores #**1**-#**6**.

S2. Fluorescence Lifetime Decay Curves:

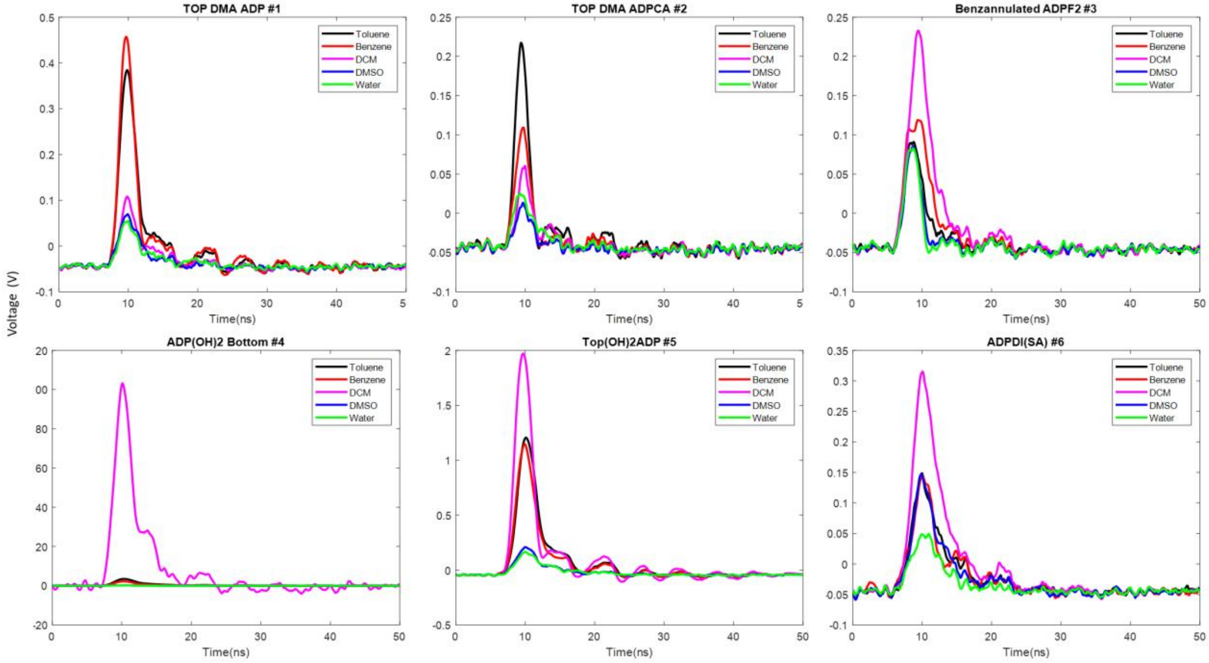

Figure S2. Fluorescence lifetime decay curves for fluorophores in different solvents.

S3. Effect of filtration and dilution on LCST*:* The effect of filtration and dilution on the LCST was studied using the Pluronic F-127 loaded with fluorophore **4** (**S3**). Filtration in the following experiment refers to (10,000 MWCO) Amicon ultra centrifugation filters. As discussed previously, all samples were synthesized at 5% (w/v) of the Pluronic initially, and 1% and 0.2% samples were prepared by dilution with D.I. water. We compared the switching behavior of filtered 1% and 0.2% samples with the unfiltered samples. As evident from the switching curves of **S3-a** for the non-filtered samples, dilution from 1% to 0.2% increased the LCST for about 3 °C from 22 to 25 °C. However, with filtration, the two samples had similar LCST at 25 °C (**S3-b**). This may be understood as follows. Without filtration, the monomers that did not form nanoparticles were still in the solution and contributed to the hydrophobicity of the microenvironment and hence LCST. This is because higher hydrophobicity is concurrent with a lower LCST. The filtration, on the other hand, disposes of most of these monomers, and the polarity of the environment does not change considerably upon dilution, and so the LCST remains the same.

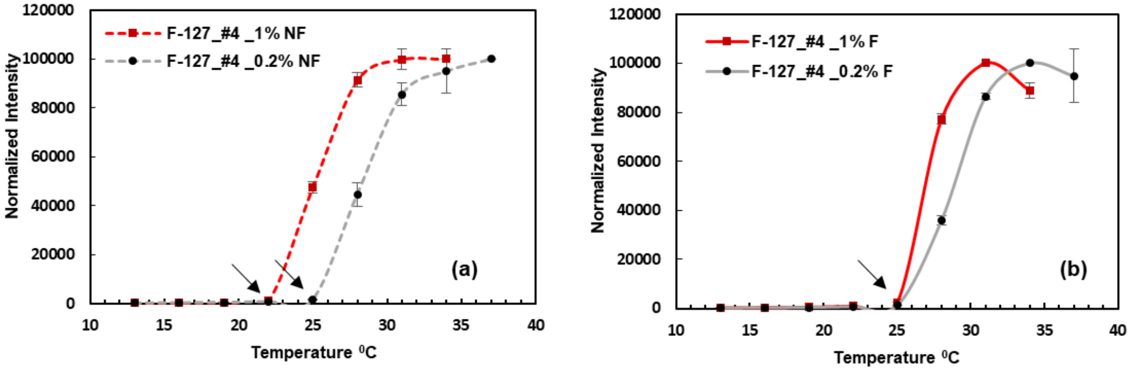

S3. (a)The LCST of 1% non-filtered F-127 Pluronic nanoparticles loaded with fluorophore **4** is about 3° C lower than that of the 0.2% sample (b). 1% and 0.2% samples have the same LCST after filtration (Values are normalized).

S4. **Compound 2 (Top DMAADPCA) Synthesis Procedure**:

Synthesis of Compound 1a: Compound 1[1] (150 mg, 0.25 mmol) was dissolved in (30 cm3) dry CH2Cl2 and stirred under argon for 15 min. Then AlCl3 (171.2 mg, 1.28 mmol) was added and the solution was further stirred for 30 min before the addition of 3, 4-dihydroxybenzaldehyde (177.3 mg, 1.28 mmol). The mixture was stirred for 30 min and the reaction mixture was flushed through deactivated basic alumina column with CH2Cl2 as eluent. The crude product was further purified by column chromatography (silica) with Ethylacetate/CH2Cl2 (1:4) to give compound 1a: Yield 115 mg (66 %); 1H NMR (400 MHz, CDCl3) δ= 9.50 (s, 1H), 8.12 -8.08 (d, 4H), 7.72 – 7.68 (d, 1H), 7.38–7.35 (d, 1H), 7.26-7.22 (d, 4H),7.94–6.82(m, 10H), 6.60 (s, 2H), 6.24–6.22 (d, 1H), 3.10 (s, 12H).

Synthesis of Compound 2 (Top DMAADPCA): compound 1a (115 mg, 0.16 mmol) was dissolved in (50 cm3) acetonitrile, and cyanoacetic acid was added (40.82 mg, 0.48 mmol) followed by the addition of piperidine (0.3 ml, 3.36 mmol), and the resulting mixture was refluxed overnight under nitrogen. Then the solvent was removed under reduced pressure. The residue was purified by column chromatography on silica gel with CH2Cl2: methanol (4:1) to give the compound 2. Yield 59 mg (49 %); 1H NMR (400 MHz, CDCl3) δ= 8.12 -8.08 (d, 4H), 7.98 (s, 1H), 7.26-7.22 (d, 4H), 6.90–6.98(m, 8H), 6.78-6,74 (d, 4H), 6.58 (s, 2H), 6.00–6.02 (d, 1H), 3.10 (s, 12H); 13C NMR (500 MHz, CDCl3) δ =157, 152, 144, 132.5, 132, 129.5, 129, 128, 127.5, 116, 113.5, 112, 68, 42, 43, 32; MALDI-Mass(+) ; m/z: $C_{46}H_{37}BN_6O_4$ calcd: 748.30, found 748.29

^{1}H and ^{13}C NMR Spectra of Compound 2

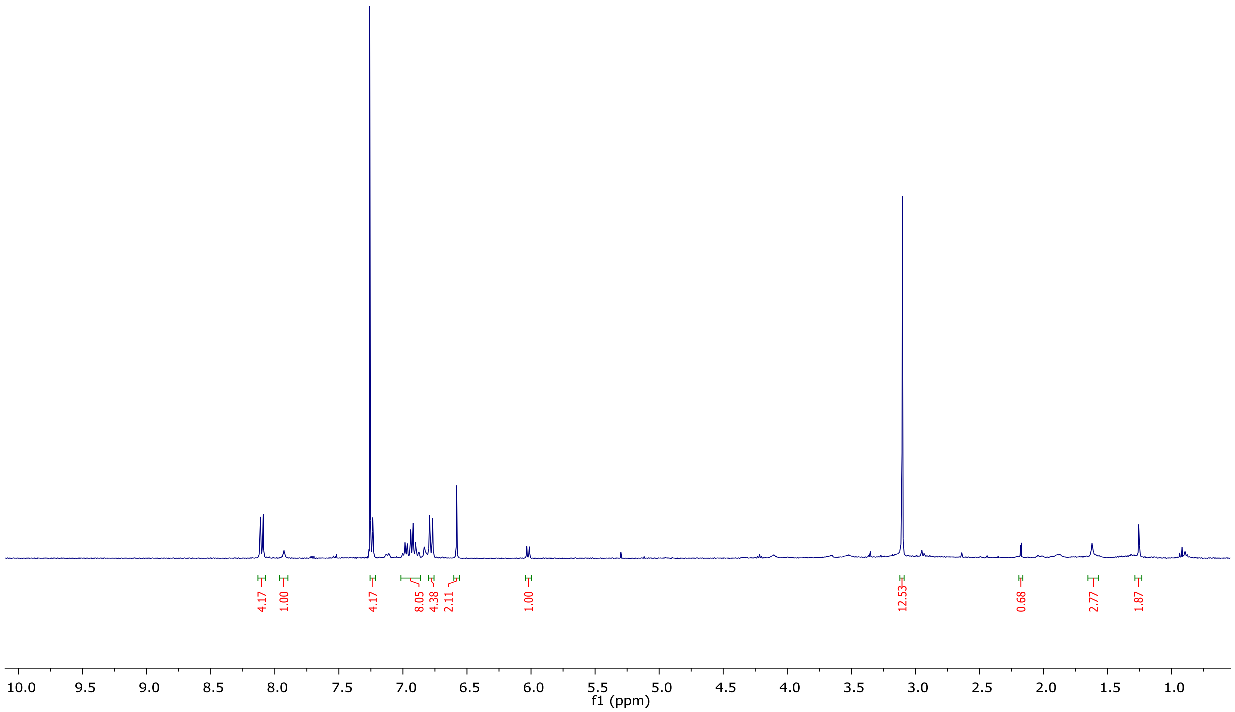

^{1}H NMR spectra for Compound 2 in $CDCl_3$ recorded with 400 MHz instrument.

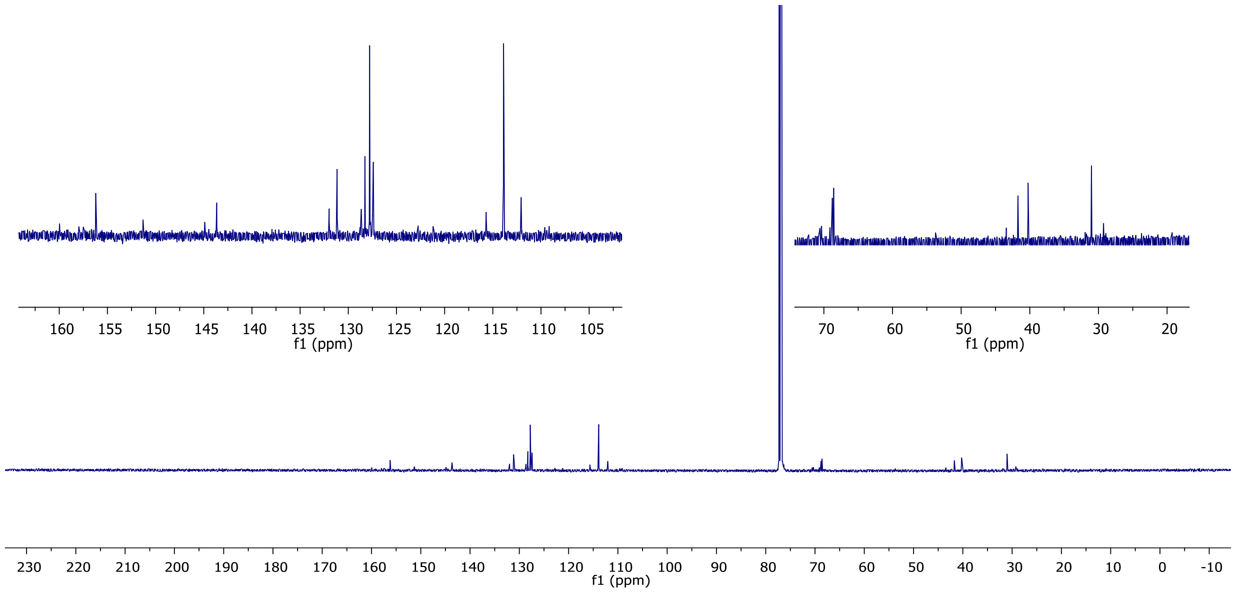

^{13}C NMR spectra for Compound 2 in $CDCl_3$ recorded with 400 MHz instrument (Insert is the zoomed version of the spectra).

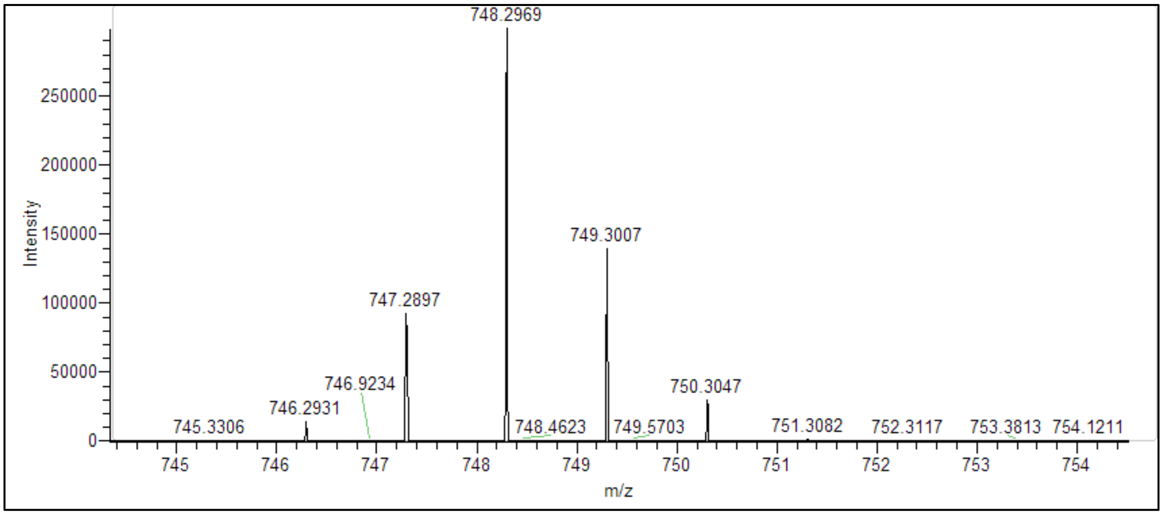

MALDI-MS spectra for Compound 2.

(1) Killoran, J.; McDonnell, S. O.; Gallagher, J. F.; O'Shea, D. F. A Substituted BF2-Chelated Tetraarylazadipyrromethene as an Intrinsic Dual Chemosensor in the 650-850 Nm Spectral Range. *New J. Chem.* **2008**, *32* (3), 483–489. https://doi.org/10.1039/b713020a.
